# Setmelanotide-mediated MC4R activation improves hypothalamic obesity via CaMKK2/AMPK pathways

**DOI:** 10.3389/fphar.2025.1730786

**Published:** 2026-01-12

**Authors:** Junjie Peng, Yichao Ou, Mingfeng Zhou, Xingqin Wang, Xi’an Zhang, Hao Long, Guangsen Wu, Mengjie Che, Kai Li, Le Yang, Zhu Zhang, Ken Kin Lam Yung, Songtao Qi, Zhanpeng Feng

**Affiliations:** 1 Department of Neurosurgery, Institute of Brain Diseases, Nanfang Hospital, Southern Medical University, Guangzhou, China; 2 Teaching and Research Division, School of Chinese Medicine, Hong Kong Baptist University, Hong Kong SAR, China; 3 Department of Science and Environmental Studies, the Education University of Hong Kong, Hong Kong SAR, China; 4 Ganzhou Hospital-Nanfang Hospital, Southern Medical University (Ganzhou People’s Hospital), Ganzhou, China

**Keywords:** CaMKK2/AMPK signaling, GPCRs, hypothalamic obesity, melanocortin 4 receptor, neuropharmacology, setmelanotide

## Abstract

Hypothalamic obesity (HO) is a disabling disease caused by central nervous system (CNS) damage due to neurosurgery, trauma, or tumors, especially in hypothalamus. The pathological mechanism of its neural circuits is still unclear, and there is currently no corresponding drug due to the complex etiology. G protein-coupled receptors (GPCRs) regulate neural function in many CNS diseases. Among them, melanocortin 4 receptor (MC4R) regulate metabolism and appetite in the hypothalamus. Setmelanotide, an MC4R agonist, has demonstrated anti-obesity effects in genetic forms of obesity; however, its efficacy and mechanisms in HO remain unexplored. This study explored the potential of treating HO by setmelanotide-targeted activation of MC4R in the paraventricular nucleus (PVN). We established a rat hypothalamic injury model to replicate human HO symptoms, such as hyperphagia (50% increase in food intake), elevated Lee index, and more than 25% weight gain. Immunofluorescence and immunoblot analysis showed that HO disrupted the PVN neuropeptides, leading to the inhibition of MC4R via calmodulin-dependent protein kinase kinase 2 (CaMKK2) and AMP-activated protein kinase (AMPK) signaling. Crucially, administration of setmelanotide restored CaMKK2/AMPK activity, reactivated MC4R neurons, and normalized appetite and feeding behavior during fasting-refeeding and the long-term treatment of obese rats (60% reduction in food intake), ultimately reversing obesity (23% weight loss). These findings underscore the critical role of MC4R dysfunction in hypothalamic injury and highlight the strategies to pharmacologically activate MC4R via CaMKK2/AMPK signaling to restore metabolic homeostasis, proposing a translatable therapeutic agent to manage obesity caused by CNS injury.

## Graphical Abstract

**Figure:**
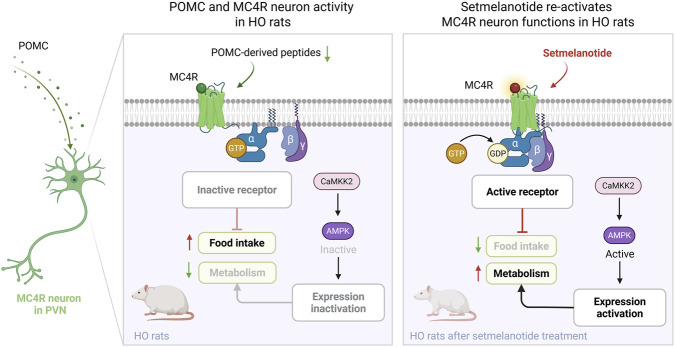

## 1 Introduction

Obesity is a growing global health problem characterized by excess body weight and associated metabolic disorders (Yang et al., 2022). While most cases of obesity result from high-fat diets or genetic predispositions (Deshpande et al., 2020), hypothalamic obesity (HO) arises uniquely from central nervous system (CNS) damage—including trauma, tumors, or neurosurgery—that impairs hypothalamic neurons (Roth and McCormack, 2024). HO is marked by hyperphagia and severe, treatment-resistant weight gain, affecting up to 50% of patients with hypothalamic tumors (van Iersel et al., 2019; Witte et al., 2024; Müller, 2025a). Current therapies predominantly target diet or genetics, leaving a significant knowledge gap in managing CNS-driven obesity syndromes such as HO.

The hypothalamus is a key regulation center for energy metabolism (Park and Ahima, 2015), with key nuclei such as the arcuate nucleus (ARC) and ventromedial hypothalamus (VMH) orchestrating appetite control. Within the ARC, anorexigenic POMC neurons and orexigenic NPY/AgRP neurons respond to peripheral signals (leptin, insulin, gut peptides), sending projections to the paraventricular nucleus (PVN). Here, melanocortin 4 receptor (MC4R)-expressing neurons integrate these inputs to drive satiety and autonomic regulation of energy expenditure (Deem et al., 2022; Gao and Horvath, 2008). Lesions of ARC/VMH disrupt this circuitry, blunting POMC-PVN MC4R signaling and leading to persistent hyperphagia, impaired autonomic output, and the clinical syndrome of HO.

Targeting hypothalamic circuits, especially the PVN MC4R axis, offers a promising avenue for HO treatment. G protein-coupled receptors (GPCRs) such as MC4R play central roles in CNS homeostasis (Ramírez and Claret, 2015; Sayar-Atasoy et al., 2023). While MC4R agonists like setmelanotide have demonstrated efficacy in rare monogenic obesity syndromes involving MC4R mutations (Fatima et al., 2022), their therapeutic potential for acquired HO remains unexplored. Despite setmelanotide’s clinical success, its effects in HO models with hypothalamic injury and neuronal loss are poorly understood, presenting a critical gap for translational research.

Setmelanotide (formerly known as RM-493 or BIM-22493) is a cyclic eight-amino acid peptide MC4R agonist (Al Musaimi et al., 2021; Falls and Zhang, 2019). It provides potent anti-obesity effects with a favorable safety profile, in contrast to first-generation MC4R agonists that were limited by cardiovascular side effects (Haqq et al., 2022). Yet, to date, there has been no systematic study of setmelanotide’s efficacy or pharmacological mechanisms in hypothalamic obesity animal models.

Therefore, this study evaluates the therapeutic response to setmelanotide in a rat model of HO induced by bilateral ARC/VMH lesions, focusing on PVN MC4R activity and downstream CaMKK2/AMPK signaling pathways. Our data demonstrate that setmelanotide reactivates PVN MC4R signaling, suppresses appetite, and reverses metabolic pathology in HO, providing mechanistic insight and highlighting the urgent need for innovative therapies for CNS-related obesity.

## 2 Materials and methods

### 2.1 Animals and experimental groups

Male Sprague–Dawley rats (6–8 weeks, 170–210 g) were housed in independent cages in a temperature-controlled room with a daily light and dark cycle. Food and water were provided *ad libitum* except during the fasting–refeeding test. Surgical procedures, drug administration, and outcome assessments (body weight, food intake, histology, and molecular assays) were performed by investigators blinded to group allocation. All procedures followed our institutional guidelines and were approved by the ethics committee of Nanfang Hospital, Southern Medical University.

Animals were randomized into groups using a computer-generated randomization sequence, prepared and assigned by a technician not involved in subsequent experiments. For each experiment, animals were assigned to: sham surgery (n = 12), lesion surgery (n = 15), refeeding lesion rats (), short-term setmelanotide treatment following lesion (n = 14), short-term saline treatment (n = 13), long-term saline (n = 13), long-term setmelanotide (n = 14). Sample sizes were determined based on prior literature and power calculation to ensure sufficient statistical power (Song et al., 2022).

### 2.2 Electrical lesion surgery

Bilateral ARC/VMH lesions were induced under isoflurane anesthesia (5% induction, 2% maintenance; Reward small animal gas device) and stereotaxic guidance. Using coordinates relative to bregma (2.6 mm posterior, 0.6 mm lateral to midline, 9.6 mm ventral), a 1-mm-thick insulated stainless-steel electrode (0.45-mm tip) was lowered bilaterally into the hypothalamus (Song et al., 2022). A cathodic current of 1.2 mA was delivered for 25 s using a constant power supply (53500, UGO Basile, Italy). Sham-operated controls underwent the same procedure without current application.

Regions and extent of hypothalamic damage were confirmed in all animals postmortem via immunohistochemical staining. Only animals with bilateral, well-confined ARC/VMH lesions, and no damage to adjacent hypothalamic nuclei, were included. Exclusion criteria comprised off-target or unilateral lesions, overt perioperative complications, significant tissue loss, or failure to recover baseline feeding within 72 h post-surgery.

### 2.3 Evaluation of obesity

All animals were housed in independent cages, and body weight and food intake were recorded weekly. The linear distance between the tip of the rat’s nose and the anus was recorded at the end of 4^th^ week as the body length of the rat, and the evaluation of obesity was indicated by the Lee index (Lee index = ∛wt (g)/length (cm) × 1,000) and the perirenal adipose tissue (PRAT) weight (Jasim, 2021; Baumann et al., 2025).

### 2.4 Fasting-refeeding and setmelanotide preparation

In the short term of fasting-refeeding test (Igarashi et al., 2023), rats were fasting for 24 h with water available, and then the food consumption was recorded every half hour during the total 2 h refeeding process.

Setmelanotide (MCE, CAS No. HY-19870) was freshly dissolved in sterile saline before use and kept at 4 °C, shielded from light, for no longer than 24 h. In the short term, after fasting for 24 h with water available, rats of the setmelanotide (SET) group were administered with setmelanotide (0.05 mg/kg body weight) by intraperitoneal injection (I.P.) according the previous studies (Pressley et al., 2022; Lazare et al., 2024). The dose for each injection was calculated based on the animal’s body weight measured immediately prior to injection. Saline rats were injected with saline. 0.5 h later the refed process began, and the food consumption was recorded every half hour during the total 2 h refeeding process.

### 2.5 Long-term setmelanotide treatment

In the long-term treatment paradigm, starting on postoperative day 21, rats were singly housed with *ad libitum* access to chow and water and received once-daily I.P. of either vehicle (saline) or setmelanotide (0.05 mg/kg). The injection volume and dose were recalculated and adjusted for each rat’s current body weight prior to every injection throughout the experiment. All injections were performed at the same time each day. Food intake was recorded at 1 h and 24 h after each injection.

### 2.6 Histology and exclusion criteria

Rats were deeply anesthetized with 1% sodium pentobarbital, frozen with normal saline, and then instilled with 4% paraformaldehyde. Then, the brain tissues were immersed in 4% paraformaldehyde for 48 h and transferred to 70%–100% ethanol. Individual tissues for brain biopsy material were placed in processing cassettes, dehydrated through a serial alcohol gradient, and embedded in paraffin wax blocks. Before immunostaining, 3-µm-thick brain tissue sections were dewaxed in xylene, rehydrated through decreasing concentrations of ethanol, and washed in PBS. In addition, the samples were then stained with hematoxylin and eosin. After staining, the sections were dehydrated with increasing concentrations of ethanol and xylene, and sealed with resin. The images were captured with a microscope (Olympus, BX63). Only animals with confirmed bilateral, well-confined ARC/VMH lesions and no adjacent tissue damage were included.

### 2.7 Immunofluorescence staining

For immunofluorescence staining of coronal frozen brain sections, the sections were rinsed with PBS, followed by blocking with 5% nonspecific antigen goat serum for 1 h at 37 °C. Then, the sections were incubated overnight at 4 °C with specific primary antibodies. The next day, after rinsing three times with PBS containing 0.5% Triton X-100, the sections were incubated for 1 h at 37 °C with the corresponding secondary antibodies conjugated with Alexa 488 or Alexa 594 (Thermo Fisher Scientific, United States). The primary and secondary antibodies were diluted in PBS containing 5% normal goat serum and 0.2% Triton X-100. After incubation with secondary antibodies, the sections were mounted on glass slides, and cover glasses were mounted in mounting medium. Fluorescence images were captured with a confocal microscope (LSM880, Zeiss, Germany). For cell number counted by ImageJ, the data are presented as the number of cells/mm^2^. For double staining analysis, the data are presented as the ratio of the number of double-positive cells to the number of single-positive cells.

### 2.8 Protein extraction, sodium dodecyl sulfate–polyacrylamide gel electrophoresis (SDS–PAGE) and Western blotting

Hypothalamic PVN tissues were harvested under a microscope, and washed with PBS three times and lysed with RIPA buffer (50 mmol/L Tris-HCl pH 8.0, 1 mmol/L EDTA pH 8.0, 5 mmol/L DTT, 2% SDS) containing protease inhibitor and phosphoric acid protease inhibitor at 4 °C for 30 min. The protein concentration was determined using a BCA assay kit (Beyotime Inc., China), after which the protein was loaded onto a 10% SDS-polyacrylamide gel and electroblotted onto polyvinylidene difluoride (PVDF) membranes (Millipore, United States). The membranes were incubated with primary antibodies against p-AMPK (1:1,000; CST), AMPK (1:1000; CST), p-CaMKK2 (1:1000; CST), CaMKK2 (1:1000; PTG) and GAPDH (1:5000, PTG) at 4 °C overnight. Subsequently, the membranes were incubated with secondary antibodies (1:1000, ABclonal). The immunoreactive signals were visualized with enhanced chemiluminescence reagents, and images were captured with a digital camera (Pierce, Rockford, IL, United States).

### 2.9 Antibodies

The following primary antibodies were used: rabbit anti-POMC (ab94446; Abcam); mouse anti-POMC (66358-1-Ig; Proteintech Group); rabbit anti-MC4R (GTX31382; Genetex); mouse anti-cFos antibody (GTX60996; Genetex); rabbit anti-p-AMPK (2535; CST), mouse anti-AMPK (66536-1-Ig, Proteintech Group), rabbit anti-p-CaMKK2 (12716; CST), rabbit anti-CaMKK2 (13730-1-AP; Proteintech Group); and rabbit anti-GAPDH (10494-1-AP, Proteintech Group). The following secondary antibodies were used: goat anti-rabbit 488 fluorescent antibody (A-11008; Thermo Fisher Scientific); goat anti-mouse 594 fluorescent antibody (A-11005; Thermo Fisher Scientific); and anti-rabbit IgG-HRP antibody (1:1000, AS014, ABclonal).

### 2.10 Statistical analysis

All statistical analyses were performed using GraphPad Prism 10.0 software. Data were represented as mean ± SEM and analyzed with a two-tailed Student’s t*-*test between two groups, and one-way ANOVA followed by LSD correction between four groups. Exact sample sizes for each analysis are reported in figure captions. *P* < 0.05 was considered to indicate significance.

## 3 Results

### 3.1 Hypothalamic obesity induced by hyperphagia after ARC/VMH lesion

**FIGURE 1:**
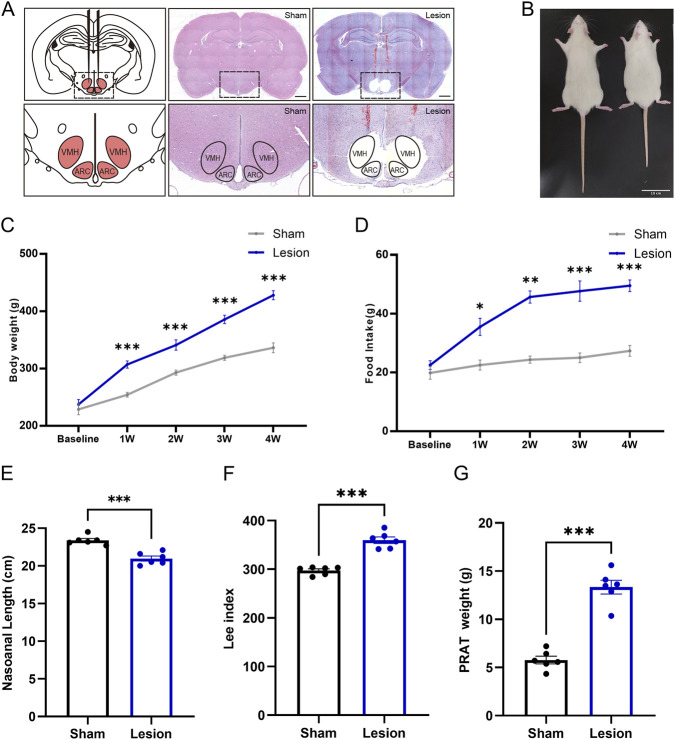
Induction of obesity via ARC/VMH lesion surgery. **(A)** Schematic overview of the mediobasal hypothalamus and placement of electrodes to target the ARC and VMH. Coronal brain sections (−2.6 mm from the bregma) from sham and lesion rats; scale bar, 1 mm. **(B–D)** Figure, body weight and food intake of the baseline and 4 weeks after surgery in sham-operated vs. lesion-operated group. n = 6. **(E–G)** Nasoanal length, Lee index and PRAT weight of sham and lesion rats at 4 weeks post-surgery. n = 6. *P < 0.05, **P < 0.01, ***P < 0.001.

Hypothalamic lesions often damage the ARC/VMH area and result in HO (Song et al., 2022; King, 2006; Joly-Amado et al., 2014). To investigate the effects of hypothalamic injury on feeding behavior and metabolic function, we induced HO by targeted electrolytic injury of ARC/VMH in SD rats, with representative coronal sections confirming lesion localization (Figure 1A). A total of 7.35% of animals were excluded due to off-target lesions, perioperative complications, or failure to recover baseline feeding; only animals with bilateral, well-confined ARC/VMH lesions were included in the analyses.

Body weight analysis showed no difference in the beginning between the two groups; however, 4 weeks after injury, the body weight of the lesion rats was 27% higher than the sham group, which were considered to be obesity in animal research (Figures 1B,C). On the first week, the food intake of lesioned rats increased significantly and this hyperphagic response persisted, and by the 4^th^ week, the food intake of lesion rats was reached to 1.96 times than that of the sham group (Figure 1D). In addition, the nasal anal length of lesioned rats was significantly shortened, and the Lee index and PRAT weight were significantly increased, further confirming the occurrence of HO after lesion (Figures 1E–G).

These findings demonstrate that ARC/VMH lesions effectively model HO, characterized by increased appetite and body weight. This surgery-induced obesity model provides a platform for evaluating potential pharmacological interventions.

### 3.2 Decreased activation of PVN MC4R in HO rats

**FIGURE 2:**
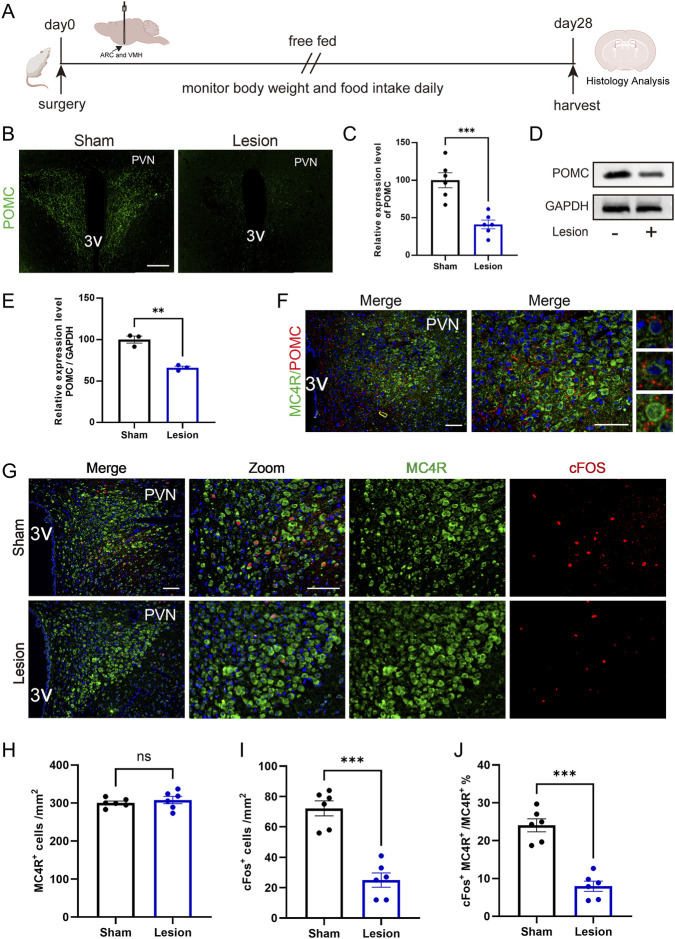
Reduced activation of PVN MC4R in HO rats. **(A)** Schematic illustration of surgery protocol in rats. **(B,C)** Immunofluorescence of PVN POMC fiber density. Scale bar, 200 μm. n = 6. **(D,E)** Protein expression levels of POMC. GAPDH was used as a loading control. n = 3. **(F)** Immunofluorescence of POMC and MC4R; MC4R (green), POMC (red), DAPI (blue). Scale bars: 100 μm (low magnification), 50 μm (high magnification). **(G–J)** Immunofluorescence of MC4R and cFos; scale bars: 100 μm (low magnification), 50 μm (high magnification), MC4R (green), cFos (red), DAPI (blue). Quantified activation of MC4R. n = 6. **P < 0.01, ***P < 0.001.

In the PVN, POMC and MC4R neurons play a key role in inhibiting food intake and maintaining metabolic balance (Guo et al., 2024; Talbi et al., 2025). To explore the underlying mechanism, we first used immunofluorescence to detect POMC expression in the PVN of HO rats. The immunofluorescence showed that POMC fiber density was significantly decreased compared to the sham group (Figures 2A–C). Western blot analysis further confirmed the decreased POMC protein expression in HO rats (Figures 2D,E).

Given that MC4R neurons are the primary recipients of POMC within the PVN (Griffin et al., 2024), we further performed double immunofluorescence staining, which revealed the distribution and contact of POMC fiber density with MC4R neurons (Figure 2F). Double immunofluorescence staining of cFos, a classical marker for neuron activation, confirmed that activation of MC4R neurons is significantly reduced in HO rats (Figures 2G–J). Reduced activation of MC4R is associated with impaired appetite suppression and the development of HO in rats.

**FIGURE 3:**
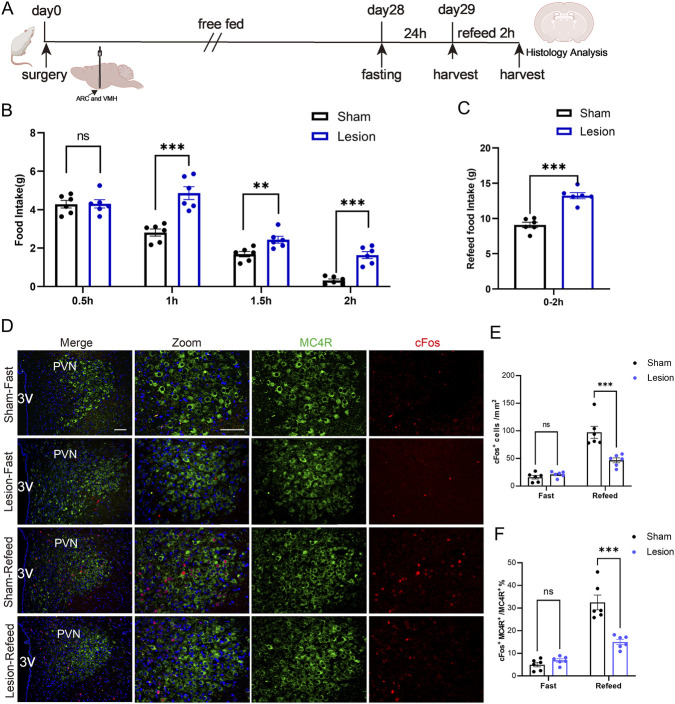
Reduced activation of PVN MC4R following fasting-refeeding treatment in HO rats. **(A)** Schematic illustration of the fasting-refeeding treatment protocol. **(B,C)** Food intake at 0.5-h intervals and total intake during 2 h of refeeding and total food intake during refeeding. n = 6. **(D–F)** Immunofluorescence of MC4R neurons and cFos in PVN; scale bars: 100 μm (low magnification), 50 μm (high magnification), MC4R (green), cFos (red), DAPI (blue). Quantified activation of MC4R. n = 6. **P < 0.01, ***P < 0.001.

Refeeding, the process of reintroducing food after fasting, plays a critical role in restoring metabolic balance and regulating appetite mechanisms in obesity research (Imoto et al., 2021). We then examined the role of MC4R neurons in appetite suppression during the fasting-refeeding treatment. Rats were fasted for 24 h at 4 weeks post-surgery and then refeed for 2 h (Figure 3A). Although there was no difference in food intake between two groups in the initial 0.5 h, HO rats showed impaired appetite inhibition in the following 0.5h–2 h, and resulting in increased food consumption compared with sham rats in total refeeding time (Figures 3B,C).

During the fasting period, there was no difference in MC4R activation between the two groups. Interestingly, after refeeding, the activation of MC4R neurons in HO rats was significantly reduced compared with sham rats (Figures 3D–F).

These findings indicate that reduced MC4R activation in HO rats, especially during refeeding, weakens the ability to suppress appetite, leading to short-term hyperphagia.

### 3.3 PVN MC4R-mediated appetite suppression via CaMKK2/AMPK signaling

MC4R neurons were reported to be associated with the regulation of the AMP-activated protein kinase (AMPK) pathway, which plays a critical role in various metabolic processes (Zhang et al., 2017). Recent studies have highlighted the involvement of calmodulin-dependent protein kinase kinase 2 (CaMKK2) in enhancing the phosphorylation and activation of AMPK, thereby influencing metabolic regulation (Chen et al., 2023).

**FIGURE 4:**
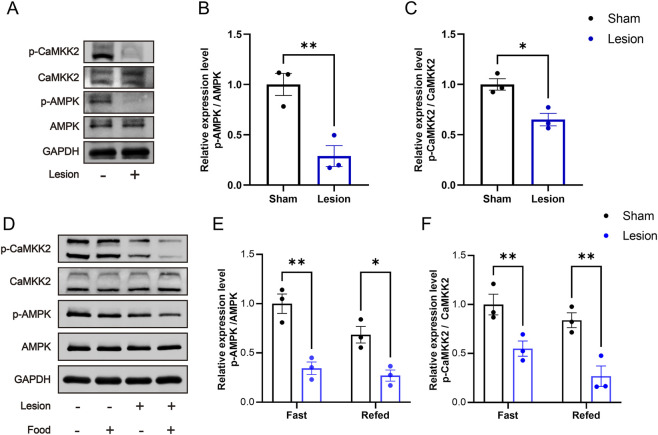
Downregulation of p-AMPK/p-CaMKK2 in HO rats and after the fasting-refeeding treatment. **(A–C)** Western blot analysis and quantification of CaMKK2 and AMPK pathway markers at 4 weeks post-surgery. n = 3. **(D–F)** Protein expression levels and quantification of CaMKK2 and AMPK in the fasting-refeeding tests. GAPDH was used as a loading control. n = 3. *P < 0.05, **P < 0.01.

Western blot analysis demonstrated a significant decrease in the activation of the AMPK and CaMKK2 signaling pathway in lesion rats compared to sham rats (Figures 4A–C). Furthermore, to evaluate these signaling pathways within the appetite inhibition in HO rats, we examined the CaMKK2/AMPK expression in fasting-refeeding treated rats. The results showed that refeeding significantly reduced pAMPK in controls, whereas pCaMKK2 did not differ between states. In lesioned rats, pCaMKK2 was lower than controls under both fasting and refeeding and remained non-responsive to refeeding; the refeeding-induced decrease in pAMPK was blunted relative to controls (Figures 4D–F), which indicated a major role of AMPK signaling pathway in the appetite regulation post-lesion surgery.

In summary, the above data underscore that AMPK pathway and MC4R exhibit consistent effects in modulating appetite inhibition in HO rats, highlighting their interconnected roles in this regulatory mechanism and offering potential mechanisms for managing HO.

### 3.4 Setmelanotide reserves hyperphagia via CaMKK2/AMPK pathways in the refeeding process

Previous findings suggest that activation of MC4R contributes to decreased appetite inhibition and HO in rats through the CaMKK2/AMPK pathways. To treat obesity in this rodent model, the agonist of MC4R, setmelanotide was used in HO rats.

**FIGURE 5:**
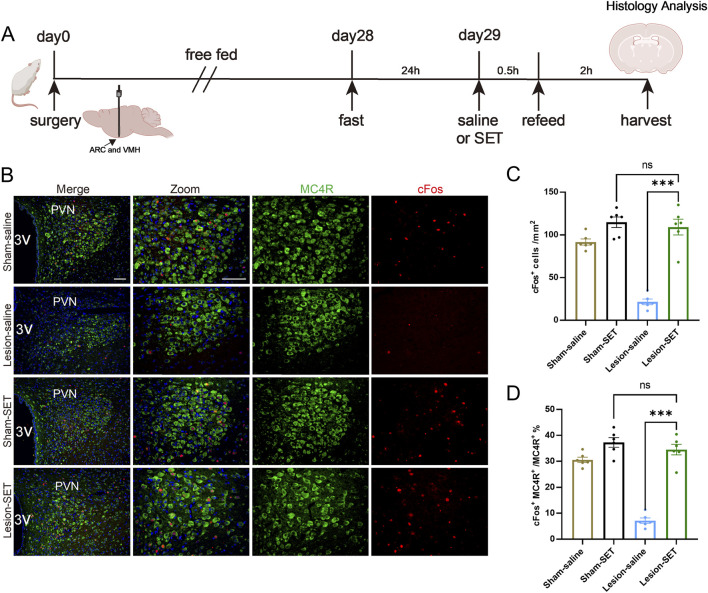
SET activates PVN MC4R in the fasting-refeeding treatment. **(A)** Schematic illustration of saline or SET injection protocol during fasting-refeeding in rats. **(B–D)** Immunofluorescence of MC4R and cFos in PVN; scale bars: 100 μm (low magnification), 50 μm (high magnification), MC4R (green), cFos (red), DAPI (blue). Quantified activation of MC4R. n = 6. **P < 0.01, ***P < 0.001.

To verify the role of setmelanotide in suppressing appetite, rats were treated with setmelanotide (Figure 5A). After refeeding, the activation ratio of MC4R in lesion-SET rats was increased, significantly higher than the lesion-saline rats (Figures 5B–D).

**FIGURE 6:**
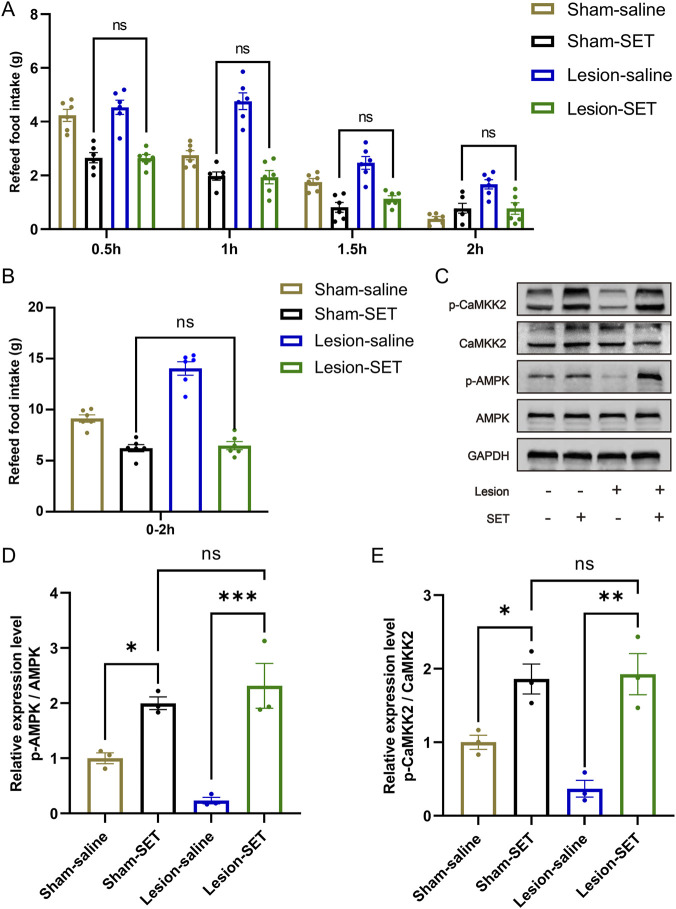
SET activates PVN MC4R via CaMKK2/AMPK pathways to restore appetite control. **(A,B)** Food intake at 0.5-h intervals and total intake during 2 h of refeeding following saline/SET injection and total food intake during refeeding. n = 6. **(C–E)** Protein expression levels and quantification of CaMKK2 and AMPK post-injection in refeeding rats. GAPDH was used as a loading control. n = 3. *P < 0.05, **P < 0.01, ***P < 0.001.

During the refeeding time, the food consumption was measured every 0.5 h, and results showed that lesion-SET rats were completely capable of controlling food consumption in the refeeding time, and there was no difference in total amount of food intake compared to the sham-SET rats. During the entire refeeding period, the lesion-SET group rats and sham-SET group rats showed similar food intake (Figures 6A,B), indicating that setmelanotide normalized feeding behavior to levels statistically indistinguishable from sham controls in the short term.

To delve into the mechanism of setmelanotide, we assessed the expression of CaMKK2/AMPK pathways. Immunoblotting confirmed that setmelanotide enhanced the activation of these 2 pathways after refeeding (Figures 6C–E). Furthermore, higher expression of p-CaMKK2/p-AMPK validated the effect of setmelanotide on restoring the metabolic homeostasis in HO rats.

These results highlight that in the short term, setmelanotide could normalize feeding behavior in HO rats by activating MC4R via the CaMKK2/AMPK pathways, underscoring its potential as a therapeutic agent for inhibiting hyperphagia in HO.

### 3.5 Long-term setmelanotide reserves HO via CaMKK2/AMPK pathways

**FIGURE 7:**
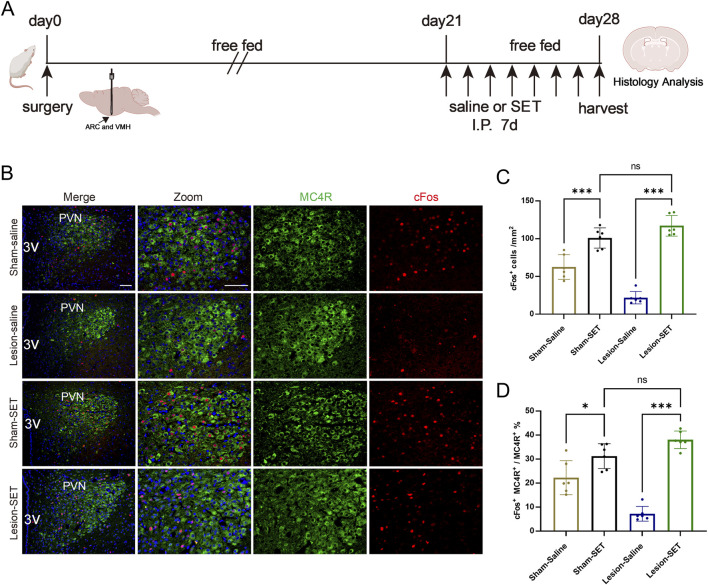
Long-term activation of PVN MC4R neurons by SET. **(A)** Schematic illustration of saline or SET injection protocol in sham/lesion rats after surgery for 3 weeks. **(B–D)** Immunofluorescence of MC4R and cFos in PVN; scale bars: 100 μm (low magnification), 50 μm (high magnification), MC4R (green), cFos (red), DAPI (blue). Quantified activation of MC4R. n = 6. **P < 0.01, ***P < 0.001.

To assess the long-term weight management of setmelanotide in HO, we treated rats with setmelanotide for 1 week (Figure 7A). Immunofluorescence analysis revealed that setmelanotide significantly strengthened the activation of MC4R. In the lesion-SET rats, the proportion of activated MC4R increased compared to the lesion-saline rats (Figures 7B–E).

**FIGURE 8:**
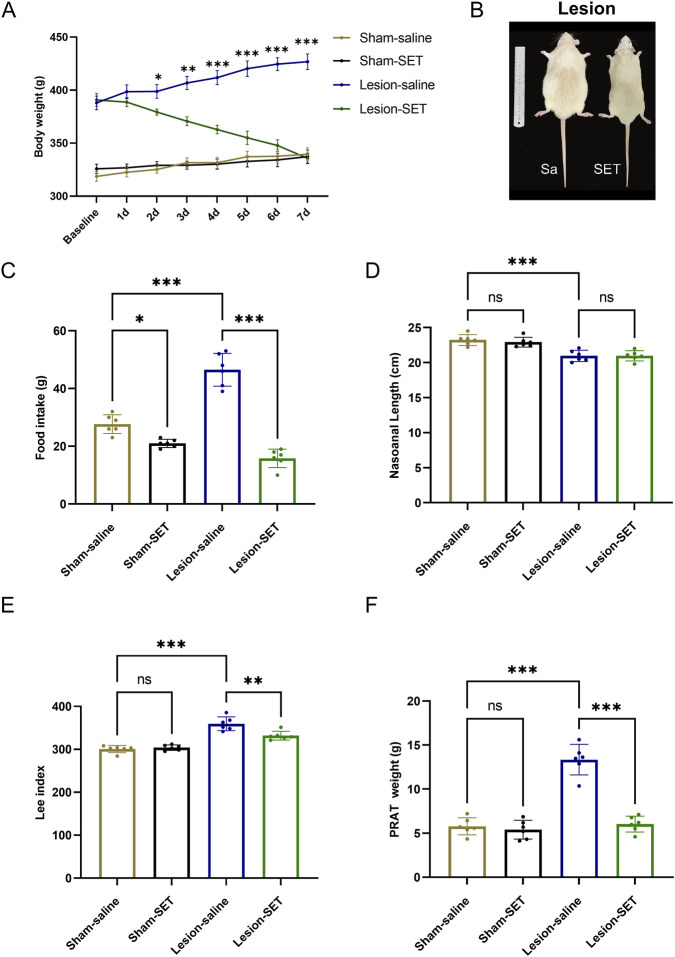
SET reduced hyperphagia and reversed obesity in HO rats. **(A–C)** Body weight, body characteristics and food intake in rats after saline or SET injection. n = 6. **(D–F)** Nasoanal length, Lee index and PRAT weight of rats after saline or SET injection. n = 6. *P < 0.05, **P < 0.01, ***P < 0.001.

Furthermore, we assessed the impact of setmelanotide in the long term. Body weight analysis showed no difference at the baseline and on the first day after administration between the lesion-saline and lesion-SET groups. Subsequently, the body weight of the lesion-SET group decreased significantly. After 1 week, body weight in the lesion-SET group was restored to levels comparable to the sham-SET group (Figures 8A,B), and food intake was significantly less than lesion-saline controls. While metabolic indices such as the Lee index and PRAT weight were significantly improved, shortened body length did not recover, indicating full reversal of some but not all parameters.

Postoperative monitoring revealed that food intake was significantly lower in lesion-SET rats compared to lesion-saline rats (Figure 8C). Additionally, while the shortened body length of lesioned rats did not recover, both the Lee index and PRAT (perirenal adipose tissue) weight were significantly reduced in lesion-SET rats versus lesion-saline rats, further confirming the anti-obesity effect of setmelanotide in HO rats (Figures 8D–F).

In summary, our findings indicate that setmelanotide treatment effectively activates PVN MC4R by upregulating the CaMKK2/AMPK pathways, thereby inhibit hyperphagia and reverse obesity in HO rats. These results underscore the potential of setmelanotide as a therapeutic strategy for managing obesity after hypothalamic injury.

## 4 Discussion

This study provides preclinical and exploratory evidence that the MC4R agonist setmelanotide may help normalize feeding behavior and energy balance in a rat model of HO, via activation of PVN MC4R neurons and engagement of CaMKK2/AMPK signaling pathways. Our results support the effectiveness of setmelanotide restoring energy homeostasis in MC4R neuron activity and HO.

HO is a subtype of obesity caused by hypothalamic injury, distanct from diet-induced or genetic obesity (Müller, 2025a; Argente et al., 2025a; Rose et al., 2018). Our ARC/VMH electrolytic lesion model partially recapitulates key features of human HO, notably hyperphagia and rapid-onset obesity following hypothalamic injury, and is especially relevant to post-surgical cases such as craniopharyngioma resection. However, human HO is clinically heterogeneous, often involving more extensive hypothalamic disruption, chronic progression, neuroendocrine and behavioral changes, as well as circadian dysregulation (Dimitri, 2022). As such, animal models can only approximate certain facets of the disease, and these differences should be considered when assessing translational applicability. Our findings suggest that pharmacological activation of MC4R neurons may help restore appetite regulation and metabolic homeostasis in contexts of hypothalamic circuit disruption, aligning with emerging evidence that MC4R agonists like setmelanotide can compensate for impaired central neuropeptide signaling (Wang et al., 2024; Kamermans et al., 2019).

Notably, the integrity of the PVN MC4R in the ARC/VMH lesion model serve as the potential target for drug treatment (Figure 2), providing mechanistic support for MC4R therapeutic strategies. In HO rats, electrolytic lesions reduced activity-dependent cFos expression in MC4R, indicating a state of functional suppression. This suppression likely resulted from disrupted input from POMC, which is damaged in our model (Figure 2). Setmelanotide is able to reactivate these neurons via the engaging of CaMKK2/AMPK signaling pathways (Figures 5, 7). Setmelanotide enhances cellular energy sensing, which synergizes with MC4R activation to suppress appetite (Hammad et al., 2022). Notably, the selectivity of setmelanotide for MC4R over other melanocortin receptors (e.g., MC3R) likely explains its reduced cardiovascular side effects compared to earlier agonists like LY2112688 (Collet et al., 2017; Clément et al., 2018). This specificity is crucial for HO patients, who often exhibit cardiovascular comorbidities (Müller, 2025b).

Our data highlight the CaMKK2/AMPK pathways as a key mediator of anti-obesity effects after setmelanotide treatment (Figure 6). In HO rats, hypothalamic CaMKK2/AMPK activity was paradoxically suppressed despite hyperphagia, suggesting an abnormality in energy metabolism and feeding behavior. Setmelanotide restored CaMKK2/AMPK phosphorylation in PVN MC4R neurons, leading to the short-term appetite suppression (Figure 3). These findings align with studies linking AMPK to MC4R function in other obesity models (Chen et al., 2018), but our work is the first to implicate CaMKK2 as the upstream kinase in this context. The CaMKK2/AMPK pathways may thus represent a druggable target for HO, particularly in patients with partial MC4R dysfunction.

While our data implicate PVN CaMKK2/AMPK signaling downstream of MC4R engagement, association rather than necessity or sufficiency. SET increased PVN MC4R-cFos and restored pCaMKK2/pAMPK in the same sampling window in which intake was normalized, but without PVN-restricted loss-of-function we cannot CaMKK2, AMPK, or both are required. Given that AMPK serves as an integrator of convergent inputs (including CaMKK2, LKB1, and phosphatases), it is biologically plausible that SET modulates AMPK and/or via CaMKK2; however, our present study does not establish pathway necessity.

Establishing causal pathway dependence will require PVN-localized perturbations. Future work will test necessity by microinjecting CaMKK2 inhibitors (e.g., STO-609) or AMPK inhibitors (e.g., Compound C) into the PVN and by implementing PVN-restricted genetic loss-of-function of CaMKK2 or AMPKα2 (e.g., Cre-dependent viral CRISPR/shRNA in MC4R neurons), together with dual-pathway perturbations to assess whether both signals are required for SET’s anorectic effects. Complementary food-withheld and pair-fed paradigms will dissociate drug effects from meal-related cues and further isolate direct MC4R-dependent signaling in PVN.

A 24-h fast was chosen because prior rat studies show robust immunometabolic shifts that enhance detection of signaling pathways (Munhoz et al., 2020). A single 24-h fast lowers basal cytokine expression in white adipose tissue of normal-weight F344 rats (Speaker et al., 2016). In our ARC/VMH-lesioned model, shorter fasts elicit smaller, more variable hypothalamic responses, reducing PVN signal reliability. We acknowledge 24 h is long and may introduce stress and circadian effects. We deliberately evaluated SET under the refeeding challenge to avoid the low-activity effect during prolonged fasting, thereby increasing the dynamic range for cFos detection and directly coupling neuronal activation to the behavioral endpoint (intake normalization). We acknowledge that we did not include a fast + SET arm to isolate drug effects; future studies will dissociate MC4R agonism from meal-related cues using food-withheld or pair-fed designs.

During refeeding after caloric restriction, lesion-SET rats displayed significantly reduced hyperphagia compared to lesion-saline rats, a phenomenon closely associated with sustained CaMKK2/AMPK activation (Figures 5, 6). pAMPK robustly tracked the fast–refed transition in control PVN, whereas pCaMKK2 did not, indicating that CaMKK2 phosphorylation is not a sensitive acute marker of feeding state at the sampling time used. This is consistent with AMPK receiving convergent inputs (CaMKK2, LKB1, and phosphatases), such that feeding can reduce pAMPK without detectable changes in pCaMKK2. In lesioned PVN, pCaMKK2 was overall reduced and non-responsive to refeeding, and the refeeding-induced suppression of pAMPK was attenuated, consistent with impaired CaMKK2/AMPK signaling. Notably, acute MC4R activation increased pCaMKK2 in both groups (Figure 6C), supporting its utility as a readout of melanocortin pathway engagement rather than an acute fast–refed marker.

Long-term administration of setmelanotide in HO rats achieved weight loss and improved metabolic parameters (reduced Lee index and perirenal adipose tissue mass), highlighting that sustained MC4R activation is critical for long-term efficacy. This shows that setmelanotide not only acutely suppresses appetite during refeeding but also normalizes adaptive feeding responses during metabolic recovery. Clinically, post-surgical hypothalamic injury patients often face weight regain triggered by overeat (Wu et al., 2022), and our results provide experimental evidence for targeting MC4R to prevent this complication—though its durability in human obesity requires validation.

We focused mechanistic analyses on perirenal white adipose tissue because it exhibits robust sympathetic innervation and is a standard readout for centrally mediated catecholamine signaling (e.g., norepinephrine turnover, lipolysis) in hypothalamic–autonomic studies (Willows et al., 2023). This choice aligns with our emphasis on PVN-sympathetic outputs after ARC/VMH injury. While perigonadal fat is also visceral and correlates strongly with whole-body adiposity, it is more sensitive to reproductive hormonal influences, inguinal fat is subcutaneous and subject to partly distinct regulatory pathways (Neuhaus and Stenkula, 2025). Thus, PRAT was selected *a priori* to maximize sensitivity to hypothalamic sympathetic signals.

Setmelanotide is successful in the treatment of genetic obesity (e.g., POMC deficiency) (Roth, 2025), indicating its potential effect in HO. Our rodent data support its translational potential, showing the reverse of hyperphagia and obesity. At present, a multicenter phase 3 study of MC4R neuron agonists has been carried out in clinical trials (Argente et al., 2025b; Trapp and Censani, 2023). MC4R was mainly used to treat increased food intake and obesity caused by the deletion of POMC gene or LEPR gene, and it could reduce hunger and cause weight loss, and 80% of the POMC gene deletion patients and 45% of the LEPR gene deletion patients had a weight loss of at least 10%, indicating that the MC4R agonist can effectively alleviate the abnormal melanocortin pathway activation caused by the POMC gene or LEPR gene deletion in clinical practice. HO patients retain the specific expression of POMC/LEPR/MC4R in the PVN, but there are currently no large-scale clinical studies on the treatment of obesity.

However, these drugs also have some side effects that cannot be ignored. For example, the most common side effects are injection site reactions and pigmentation, as well as varying degrees of nausea and vomiting, but no patients have had any serious treatment-related reactions (Collet et al., 2017). Therefore, we speculated that the drug may also be used in clinically severely obese HO patients because it specifically activates MC4R neurons and it can effectively regulate the hyperphagia and obesity caused by hypothalamic injury.

While setmelanotide reactivated PVN MC4R, off-target effects on non-hypothalamic MC4R populations need further investigation (Haqq et al., 2022; Wang et al., 2024; Meimetis et al., 2024). Additionally, while this drug could restore metabolism, it failed to promote recovery of body length, a special effect in HO rats (Figures 8B,D), indicating limitations in its efficacy for HO patients with concurrent growth-related issues. This phenomenon may relate to MC4R’s primary role in regulating energy metabolism rather than the growth hormone axis, reflecting the limitations of single-target therapy and advocating for future combinatorial approaches targeting both metabolic and growth pathways. Although our model mimics post-surgical HO, human HO sometimes involves broader hypothalamic damage (suprachiasmatic nucleus involvement), which may require combinatorial therapies in future studies.

This study has several limitations. It was conducted exclusively in male rats over a relatively short time frame, potentially limiting generalizability to female subjects or to chronic HO. The electrolytic lesion model primarily reflects post-surgical HO, but does not encompass all mechanisms or presentations seen in human patients. Our findings should therefore be interpreted as exploratory and preliminary. Future studies should evaluate setmelanotide efficacy in models that more broadly mimic clinical hypothalamic damage, include long-term and behavioral endpoints, and ultimately require rigorous clinical trials to determine therapeutic potential and safety in human HO.

Future studies are warranted to explore the efficacy of setmelanotide in chronic settings, across sexes, and in additional behavioral domains. As this study was conducted in a rodent model, the results should be considered exploratory. Given the complexity and heterogeneity of hypothalamic damage in human HO, further studies are needed to determine if findings in this model translate to clinical populations. These future investigations should employ models with broader hypothalamic involvement, incorporate long-term assessment, and include behavioral and neuroendocrine endpoints. Large-scale clinical trials will be essential to establish efficacy and safety in diverse patient populations.

In summary, this study provides preclinical evidence that setmelanotide may be a promising therapeutic option for hypothalamic obesity, acting via MC4R activation and engagement of the CaMKK2/AMPK pathway. However, as its effects are primarily limited to anti-obesity outcomes, these findings underscore the need to consider combinatorial multi-pathway strategies to address the complexity of HO pathology.

## 5 Conclusion

In summary, this study demonstrates that setmelanotide is a promising treatment for hypothalamic obesity through a mechanism of reactivated MC4R via CaMKK2/AMPK pathway. While it’s limited to anti-obesity effects, these findings underscore the need for multi-pathway combinatorial strategies to address HO’s complex pathology.

## Data Availability

The original contributions presented in the study are included in the article/supplementary material, further inquiries can be directed to the corresponding authors.
